# Association between dietary vitamin B1 intake and cognitive function among older adults: a cross-sectional study

**DOI:** 10.1186/s12967-024-04969-3

**Published:** 2024-02-16

**Authors:** Weiai Jia, Hemei Wang, Chao Li, Jingpu Shi, Fangfang Yong, Huiqun Jia

**Affiliations:** Department of Anesthesiology, The Fourth Hospital of Hebei Medical University, NO.12, JianKang Road, Shijiazhuang, 050011 Hebei People’s Republic of China

**Keywords:** Cognitive function, Vitamin B1 intake, NHANES, Cross-sectional study

## Abstract

**Background:**

This study aims to investigate the relationship between vitamin B1 intake and cognitive function in older adults.

**Methods:**

This cross-sectional observational study utilized data from the National Health and Nutrition Examination Survey (NHANES) 2011–2014. A total of 2422 participants were included in the analysis, with dietary vitamin B1 intake being determined by averaging two 24-h dietary recalls. Cognitive function was assessed using three cognitive function tests: the Digit Symbol Substitution Test (DSST) for processing speed, the Animal Fluency Test (AFT) for executive function, a Consortium to Establish a Registry for Alzheimer's disease (CERAD) subtest for memory. Test-specific and global cognition z score was created. Multivariate linear regression models were used to explore the association between vitamin B1 and cognitive function.

**Results:**

2422 participants, aged 60 years and older, were included from NHANES across two survey cycles (2011–2014). Higher vitamin B1 intake was associated with higher DSST, AFT scores (P < 0.001) as well as the global cognition z score (P = 0.008).

In the fully adjusted model, as compared to the lowest quartile (Q1), the highest quartile (Q4) of vitamin B1 intake was related to higher DSST score (β = 2.23, 95% CI 0.79 ~ 3.67) and global cognition z sore (β = 0.09, 95% CI 0.02 ~ 0.16). The association between dietary vitamin B1 intake and cognitive function scores in US adults is linear. There was no detected significant statistical interaction between these variables.

**Conclusions:**

Increased dietary intake of vitamin B1 was associated with better cognitive function in individuals aged over 60.

**Supplementary Information:**

The online version contains supplementary material available at 10.1186/s12967-024-04969-3.

## Introduction

The prevalence of cognitive impairment is increasing due to the aging of the global population [1]. From mild cognitive impairment (MCI) to Alzheimer's disease (AD), all forms of dementia are characterized by cognitive impairment and are becoming a major global public health concern [2]. Projections indicate that the number of people with dementia will increase from 57.4 million cases globally in 2019 to 152.8 million cases in 2050 [3]. In the United States, the number of adults over 65 with clinical AD will increase from 6.07 million in 2020 to 13.85 million in 2060 [4]. Therefore, exploring protectable factors related to cognitive performance is essential for preventing the onset of cognitive impairment [5]. Identifying risks and protective factors is an effective way of early prevention strategies. Physical activity and a healthy diet are modifiable factors associated that can reduce the risk of cognitive impairment [6–8]. Additionally, a nutritious diet with adequate intake of vitamins B, D, and E can have a protective effect on cognitive performance [9–11].

Vitamin B1, also known as thiamine, is a water-soluble micronutrient that plays a crucial role in energy metabolism, neuronal function, and cognitive development. Due to its short half-life and limited body stores, a consistent dietary intake is necessary to sustain adequate tissue thiamine levels [12]. Thus, thiamine deficiency can occur at any stage of life [13]. Vitamin B1 deficiency also has been linked to various neurodegenerative disorders, including Alzheimer's disease, Parkinson's disease, and Huntington's disease [14, 15]. Nevertheless, the impact of dietary vitamin B1 intake on cognitive impairment in older adults remains uncertain. Consequently, our objective is to examine the association between dietary vitamin B1 intake and cognitive function in individuals aged 60 years and older, utilizing publicly accessible data from the National Health and Nutrition Examination Survey (NHANES) between 2011 and 2014.

## Materials and methods

### Data sources and study population

The cross-sectional study utilized NHANES data from 2011 to 2014, performed by the National Center for Health Statistics (NCHS). The NHANES program employs a stratified, multistage probability cluster sampling design to conduct annual surveys, ensuring the sampled population accurately represents the entire U.S. population. Through health interviews, sociodemographic information including age, gender, household income, and education level is collected, along with health-related lifestyle variables such as alcohol consumption, smoking habits, and physical activity. All procedures were approved by the NCHS Research Ethics Committee, and participants provided written informed consent. (http://www.cdc.gov/nchs/nhanes.htm).

For this analysis, we excluded subjects with incomplete cognitive information and participants with incomplete vitamin B1 intake questionnaires [11, 16, 17]. The final sample consisted of 2422 individuals aged 60 years and above. In this study, we followed the STROBE guidelines for reporting observational studies [18].

### Dietary vitamin B1 intake

The NHANES study employed a 24-h food recall questionnaire that was made available to all participants, enabling the collection of comprehensive data regarding the type and quantity of food consumed within the previous 24 h. All NHANES participants were eligible to participate in two 24-h dietary recall interviews, with the data collected being utilized to ascertain the daily intake of vitamin B1 for each individual. The initial dietary recall interview was conducted face-to-face at the Mobile Examination Center (MEC), while the second interview took place via telephone within a period of 3–10 days [19]. Dietary vitamin B1 intake was assessed by averaging data from two 24-h dietary recalls. Participants were grouped based on their vitamin B1 intake.

### Cognitive function

In the NHANES study, participants underwent various cognitive function tests to evaluate their memory and executive skills. The immediate and delayed verbal list learning (CERAD-IRT and CERAD-DRT) test, conducted by the Alzheimer's Disease Word Learning Registry Consortium, assesses the capacity to acquire novel linguistic [20]. The participants were required to read aloud a list of 10 unrelated terms, followed by an immediate recall of as many words as possible. Their scores from the first three tests were added. Moreover, a delayed recall test was conducted approximately 8–10 min after the commencement of the word learning trial, with a score range of 0–10.The Animal Fluency Test (AFT) measures verbal and executive abilities by asking participants to name as many animals as possible in one minute [21]. For each animal named, one point is awarded [22]. The Digit Symbol Substitution Test (DSST) is a time-constrained assessment that quantifies processing speed and executive function through the task of transcribing symbols corresponding to digits, utilizing a provided legend [23]. Participants had 2 min to match symbols to numbers in 133 boxes using keys at the top. Scores were based on correct matches, ranging from 0 to 133.

The global cognition z score calculated by averaging the four standardized scores, including DSST, AFT, CERAD-IRT, and CERAD-DRT. Standardized scores were calculated from the sample mean and standard deviation of each cognitive test score. For all the tests, higher scores indicate better cognitive performance [22, 24].

### Covariates

Multiple potential covariates were evaluated based on existing literature, including gender, age, body mass index, race, marital status, education, poverty income ratio, smoking status, alcohol status, hypertension, diabetes mellitus, history of cardiovascular disease, and stroke [11, 25, 26]. The classification of race/ethnicity included non-Hispanic white, non-Hispanic black, Mexican American, and other races. Marital status was categorized as married, cohabiting, or living alone. Educational attainment was classified into three categories: less than 9 years, 9–12 years, and more than 12 years. The poverty income ratio (PIR) was utilized to classify family income into three distinct groups: low (PIR ≤ 1.3), medium (PIR > 1.3–3.5), and high (PIR > 3.5). Smoking status was classified as follows: never smokers (smoked fewer than 100 cigarettes), current smokers, and former smokers (quit smoking after consuming more than 100 cigarettes). Similarly, alcohol status was categorized as never drinking (consumed less than 12 alcoholic drinks in a lifetime), current drinking, and former drinking (consumed at least 12 drinks in a lifetime).

### Statistical analysis

The characteristics of the study participants at baseline were compared using the Chi-square test and one-way analysis of variance. Continuous variables were presented as mean and standard deviation (SD) or median and interquartile range (IQR), while categorical variables were expressed as population proportions and percentages. In order to account for multiple comparisons, pairwise comparisons with Bonferroni correction were conducted.

Vitamin B1 intake was categorized by quartiles, with Q1 being the reference group. The multivariate linear regression explored vitamin B1 intake as both a continuous and categorical variable. β and 95% confidence interval (CI) were calculated to assess the relationship between vitamin B1 intake and cognitive function. Model 1 represented the crude model without any adjustment for variables. Model 2 was adjusted for age, gender, race/ethnicity, poverty income ratio, marital status, and education level. Model 3 was adjusted as for Model 2, additionally adjusted for body mass index, smoking status, alcohol status, hypertension, diabetes mellitus, cardiovascular disease history, and stroke. Linear trend tests were performed by treating categorical variables as continuous variables. We also conducted curve fitting of the dose–response relationship between vitamin B1 intake and cognitive test scores using the restricted cubic spline approach.

Subgroup analyses were conducted using hierarchical binary linear regression models, wherein continuous variables were converted into categorical variables according to clinical cutoffs or quantiles. Interaction tests were performed, followed by effect adjustment tests on subgroup measures, and likelihood ratio tests were subsequently conducted.

All analyses were performed with the statistical software packages R 4.1.1 and Free Statistics software versions 1.7.1. A two-sided p < 0.05 was considered statistically significant.

## Results

### Characteristics of participants at the baseline

During the 2011–2014 cycle, NHANES involved the participation of 19,931 individuals.

**Fig. 1:**
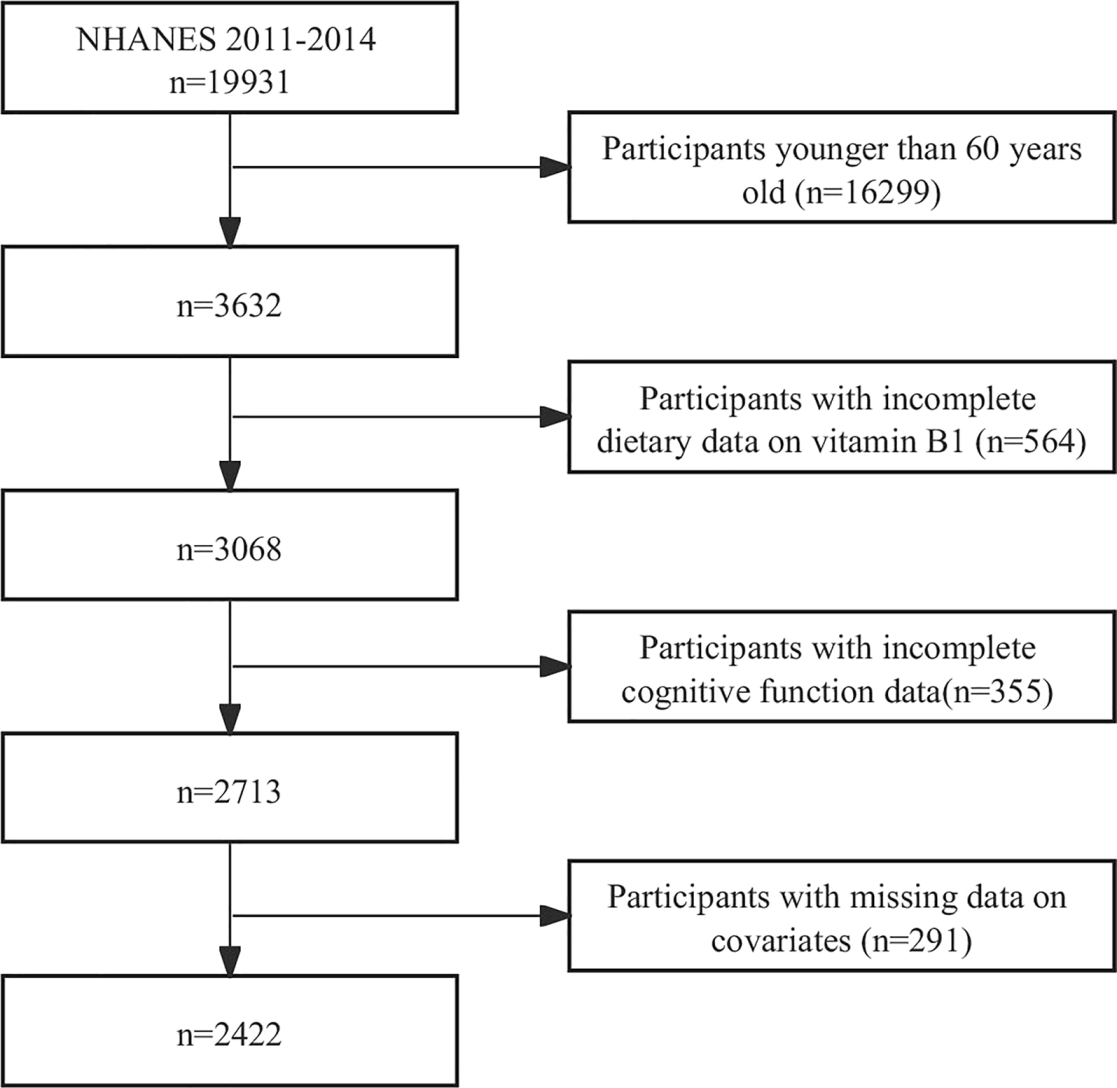
Flowchart detailing the selection process for patients included in this retrospective analysis

We excluded individuals under the age of 60 (n = 16,299), those with missing dietary data on vitamin B1 (n = 564), those with missing cognitive data (n = 355), and those with missing covariates (n = 291). As a result, a final sample of 2,422 participants was included in the study. Figure 1 illustrates the selection process in detail.

The baseline characteristics of the study participants based on their dietary vitamin B1 intake were summarized in Table 1. Quartile analysis of vitamin B1 was conducted to categorize the participants into four groups: Q1 (≤ 0.97 mg/day), Q2 (0.98 mg/day–1.33 mg/day), Q3 (1.34 mg/day–1.82 mg/day), and Q4 (> 1.82 mg/day). The mean age of the participants was 69.3 ± 6.7 years, with 1232 (50.9%) females and 1214 (50.1%) Non-Hispanic Whites. Moreover, in comparison to individuals with low vitamin B1 intake, those with higher vitamin B1 intake exhibited a greater likelihood of being Non-Hispanic White and male, as well as possessing higher family income, and lower rates of smoking. Additionally, these individuals demonstrated higher scores on the DSST, AFT, and global z score, with respective averages of 48.3 ± 15.7, 17.6 ± 5.7, and 0.10 (−0.44, 0.57) in the highest vitamin B1 intake.

**Table 1:** Characteristics of the study population, National Health and Nutrition Examination Survey (NHANES) 2011–2014 (N = 2422)

Variables	Vitamin B1 intake, mg/d					
	Total	Q1(≤ 0.97)	Q2(0.98–1.33)	Q3(1.34–1.82)	Q4(> 1.82)	*p*-Value
Number of participants	2422	606	602	608	606	
Age (years)	69.3 ± 6.7	69.5 ± 6.5	69.2 ± 6.8	69.8 ± 7.0	68.8 ± 6.6	0.077
Gender, n (%)						< 0.001
Male	1190 (49.1)	225 (37.1)	250 (41.5)	296 (48.7)	419 (69.1)	
Female	1232 (50.9)	381 (62.9)	352 (58.5)	312 (51.3)	187 (30.9)	
BMI (kg/m^2^)	29.2 ± 6.4	29.5 ± 6.3	29.2 ± 6.7	29.3 ± 6.3	28.9 ± 6.5	0.492
Race/ethnicity, n (%)						< 0.001
Non-Hispanic white	1214 (50.1)	274 (45.2)	285 (47.3)	325 (53.5)	330 (54.5)	
Non-Hispanic black	568 (23.5)	182 (30.0)	153 (25.4)	123 (20.2)	110 (18.2)	
Mexican American	206 (8.5)	40 (6.6)	44 (7.3)	60 (9.9)	62 (10.2)	
Others	434 (17.9)	110 (18.2)	120 (19.9)	100 (16.4)	104 (17.2)	
Education level (year), n (%)						0.546
< 9	245 (10.1)	69 (11.4)	61 (10.1)	58 (9.5)	57 (9.4)	
9–12	889 (36.7)	232 (38.3)	225 (37.4)	224 (36.8)	208 (34.3)	
> 12	1288 (53.2)	305 (50.3)	316 (52.5)	326 (53.6)	341 (56.3)	
Marital status, n (%)						< 0.001
Married or living with a partner	1413 (58.3)	309 (51.0)	342 (56.8)	356 (58.6)	406 (67.0)	
Living alone	1009 (41.7)	297 (49.0)	260 (43.2)	252 (41.4)	200 (33.0)	
Family income, n (%)						< 0.001
Low	704 (29.1)	213 (35.1)	176 (29.2)	173 (28.5)	142 (23.4)	
Medium	929 (38.4)	227 (37.5)	233 (38.7)	246 (40.5)	223 (36.8)	
High	789 (32.6)	166 (27.4)	193 (32.1)	189 (31.1)	241 (39.8)	
Smoking status, n (%)						< 0.001
Never	1179 (48.7)	313 (51.7)	309 (51.3)	308 (50.7)	249 (41.1)	
Former	944 (39.0)	200 (33.0)	222 (36.9)	231 (38.0)	291 (48.0)	
Current	299 (12.3)	93 (15.3)	71 (11.8)	69 (11.3)	66 (10.9)	
Alcohol status, n (%)						0.013
Never	351 (14.5)	99 (16.3)	90 (15)	96 (15.8)	66 (10.9)	
Former	688 (28.4)	189 (31.2)	173 (28.7)	166 (27.3)	160 (26.4)	
Current	1383 (57.1)	318 (52.5)	339 (56.3)	346 (56.9)	380 (62.7)	
Hypertension, n (%)						0.442
Yes	1268 (52.4)	324 (53.5)	328 (54.5)	307 (50.5)	309 (51.0)	
No	1154 (47.6)	282 (46.5)	274 (45.5)	301 (49.5)	297 (49.0)	
Diabetes, n (%)						0.134
Yes	565 (23.3)	155 (25.6)	147 (24.4)	141 (23.2)	122 (20.1)	
No	1857 (76.7)	451 (74.4)	455 (75.6)	467 (76.8)	484 (79.9)	
Coronary heart disease, n (%)						0.848
Yes	221 (9.1)	55 (9.1)	50 (8.3)	57 (9.4)	59 (9.7)	
No	2201 (90.9)	551 (90.9)	552 (91.7)	551 (90.6)	547 (90.3)	
Stroke, n (%)						0.217
Yes	161 (6.6)	50 (8.3)	42 (7.0)	34 (5.6)	35 (5.8)	
No	2261 (93.4)	556 (91.7)	560 (93)	574 (94.4)	571 (94.2)	
Cognitive score						
DSST, Mean ± SD	46.7 ± 17.0	43.9 ± 17.3	47.2 ± 17.3	47.4 ± 17.3	48.3 ± 15.7	< 0.001
AFT, Mean ± SD	16.8 ± 5.5	16.1 ± 5.3	16.7 ± 5.4	17.1 ± 5.5	17.6 ± 5.7	< 0.001
CERAD-IRT, Mean ± SD	19.1 ± 4.6	18.8 ± 4.7	19.0 ± 4.6	19.4 ± 4.4	19.1 ± 4.6	0.119
CERAD-DRT, Mean ± SD	6.0 ± 2.3	5.9 ± 2.3	6.0 ± 2.3	6.1 ± 2.2	6.1 ± 2.3	0.502
Z score, Median (IQR)	0.02 (−0.54, 0.55)	−0.03 (−0.66, 0.48)	−0.01 (−0.54, 0.53)	0.05 (−0.52, 0.63)	0.10 (−0.44, 0.57)	0.008

*%* weighted proportion, *BMI* body mass index, *DSST* Digit Symbol substation test, *AFT* Animal Fluency Test, *CERAD* Consortium to Establish a Registry for Alzheimer’s disease; *CERAD-IRT* immediate recall in CERAD trial, *CERAD-DRT* delayed recall in CERAD trial, Z score is average of the standardized scores of DSST, AFT, CERAD-IRT, CERAD-DRT, *SD* standard deviation, *IQR* interquartile range, *Q1-Q4* Quartile according to vitamin B1 intake

### Relationship between dietary vitamin B1 intake and cognitive function

Table 2 presents the findings of multivariable linear regression analysis on the association between vitamin B1 consumption and cognitive function. Our research indicated that vitamin B1 intake was positively associated with cognitive scores. In the crude model, participants in the highest quartile (Q4) groups exhibited higher cognition scores(DSST:β = 4.40, 95% CI 2.50 ~ 6.31; AFT:β = 1.48, 95% CI 0.86 ~ 2.10; Z score:β = 0.16, 95% CI 0.07 ~ 0.25) compared to the Q1 group. Moreover, as vitamin B1 intake increased, cognitive scores gradually increased (p for trend < 0.001). These associations remained statistically significant across all multivariate linear regression models, even after controlling for various covariates such as age, gender, race/ethnicity, poverty income ratio, marital status, education level, body mass index, smoking status, alcohol status, hypertension, and diabetes mellitus, cardiovascular disease history, and stroke. In the model 3, compared with Q1, participants in Q4 groups were found with higher DSST score (β = 2.23, 95% CI 0.79 ~ 3.67) and global z score (β = 0.09, 95% CI 0.02 ~ 0.16). The Q2 and Q3 groups also exhibited significantly higher DSST scores (p for trend 0.003), AFT scores (p for trend 0.048), and global z scores (p for trend 0.004) than the Q1 group. The relationship between covariates and cognitive function is shown in Additional file 1: Table S1.

**Table 2:** Multivariable linear regression to assess the association of vitamin B1 intake with cognitive function

Variable	DSST		AFT		CERAD-IRT		CERAD-DRT		Z score	
	β (95% CI)	*p*-Value	β (95% CI)	*p*-Value	β (95% CI)	*p*-Value	β (95% CI)	*p*-Value	β (95% CI)	*p*-Value
	Model 1		Model 1		Model 1		Model 1		Model 1	
VB1 (mg/day)	1.84 (0.94 ~ 2.75)	< 0.001	0.76 (0.47 ~ 1.05)	< 0.001	0.17 (−0.08 ~ 0.41)	0.188	0.11 (−0.01 ~ 0.23)	0.082	0.08 (0.04 ~ 0.12)	< 0.001
VB1 quartile										
Q1 (≤ 0.97)	1(reference)		1(reference)		1(reference)		1(reference)		1(reference)	
Q2 (0.98–1.33)	3.23 (1.32 ~ 5.14)	0.001	0.59 (−0.03 ~ 1.21)	0.062	0.15 (−0.37 ~ 0.67)	0.565	0.08 (−0.18 ~ 0.44)	0.532	0.09 (0.00 ~ 0.18)	0.041
Q3 (1.34–1.82)	3.49 (1.59 ~ 5.39)	< 0.001	1.01 (0.39 ~ 1.62)	0.001	0.61 (0.10 ~ 1.13)	0.020	0.18 (−0.08 ~ 0.19)	0.168	0.15 (0.06 ~ 0.24)	0.001
Q4 (> 1.82)	4.40 (2.50 ~ 6.31)	< 0.001	1.48 (0.86 ~ 2.10)	< 0.001	0.25 (−0.28 ~ 0.75)	0.367	0.16 (−0.10 ~ 0.42)	0.224	0.16 (0.07 ~ 0.25)	< 0.001
p for trend		< 0.001		< 0.001		0.159		0.164		< 0.001
	Model 2		Model 2		Model 2		Model 2		Model 2	
VB1 (mg/day)	0.87 (0.17 ~ 1.56)	0.015	0.27 (−0.01 ~ 0.54)	0.055	0.22 (−0.01 ~ 0.45)	0.063	0.14 (0.02 ~ 0.25)	0.023	0.05 (0.02 ~ 0.09)	0.004
VB1 quartile										
Q1 (≤ 0.97)	1(reference)		1(reference)		1(reference)		1(reference)		1(reference)	
Q2 (0.98–1.33)	2.12 (0.71 ~ 3.52)	0.003	0.31 (−0.24 ~ 0.86)	0.275	0.06 (−0.41 ~ 0.53)	0.804	0.03 (−0.21 ~ 0.26)	0.811	0.05 (−0.02 ~ 0.12)	0.151
Q3 (1.34–1.82)	2.42 (1.01 ~ 3.83)	0.001	0.54 (−0.01 ~ 1.14)	0.055	0.65 (0.18 ~ 1.12)	0.007	0.20 (−0.04 ~ 0.43)	0.105	0.12 (0.05 ~ 0.19)	0.001
Q4 (> 1.82)	2.57 (1.11 ~ 4.02)	0.001	0.57 (−0.01 ~ 1.14)	0.053	0.33 (−0.15 ~ 0.82)	0.180	0.19 (−0.05 ~ 0.44)	0.125	0.10 (0.03 ~ 0.18)	0.006
p for trend		0.001		0.035		0.040		0.059		0.001
	Model 3		Model 3		Model 3		Model 3		Model 3	
VB1 (mg/day)	0.72 (0.03 ~ 1.41)	0.040	0.26 (−0.02 ~ 0.53)	0.065	0.20 (-0.03 ~ 0.43)	0.089	0.12 (0.01 ~ 0.24)	0.037	0.05 (0.01 ~ 0.08)	0.008
VB1 quartile										
Q1 (≤ 0.97)	1(reference)		1(reference)		1(reference)		1(reference)		1(reference)	
Q2 (0.98–1.33)	1.95 (0.57 ~ 3.34)	0.006	0.30 (−0.25 ~ 0.84)	0.291	0.04 (−0.43 ~ 0.51)	0.866	0.02 (−0.22 ~ 0.25)	0.884	0.05 (−0.02 ~ 0.12)	0.192
Q3 (1.34–1.82)	2.18 (0.79 ~ 3.57)	0.002	0.51 (−0.05 ~ 1.06)	0.073	0.62 (0.15 ~ 1.09)	0.010	0.18 (−0.06 ~ 0.42)	0.137	0.11 (0.04 ~ 0.18)	0.002
Q4 (> 1.82)	2.23 (0.79 ~ 3.67)	0.002	0.53 (−0.04 ~ 1.10)	0.067	0.28 (−0.20 ~ 0.77)	0.253	0.07 (−0.01 ~ 0.14)	0.198	0.09 (0.02 ~ 0.16)	0.015
p for trend		0.003		0.048		0.063		0.100		0.004

*CI* confidence interval, *VB1* vitamin B1, *Q1-Q4* Quartile according to vitamin B1 intake, *DSST* Digit Symbol substation test, *AFT* Animal Fluency Test, *CERAD* Consortium to Establish a Registry for Alzheimer’s disease, *CERAD-IRT* immediate recall in CERAD trial, *CERAD-DRT* delayed recall in CERAD trial, Z score is average of the standardized scores of DSST, AFT, CERAD-IRT, CERAD-DRT; Model 1 was the crude model without adjustment for covariates. Model 2 was adjusted for age, gender, race/ethnicity, poverty income ratio, marital status, and education level. Model 3 was adjusted as for Model 2, additionally adjusted for body mass index, smoking status, alcohol status, hypertension, diabetes mellitus, cardiovascular disease history and stroke

### Dose–response relationships

**Fig. 2:**
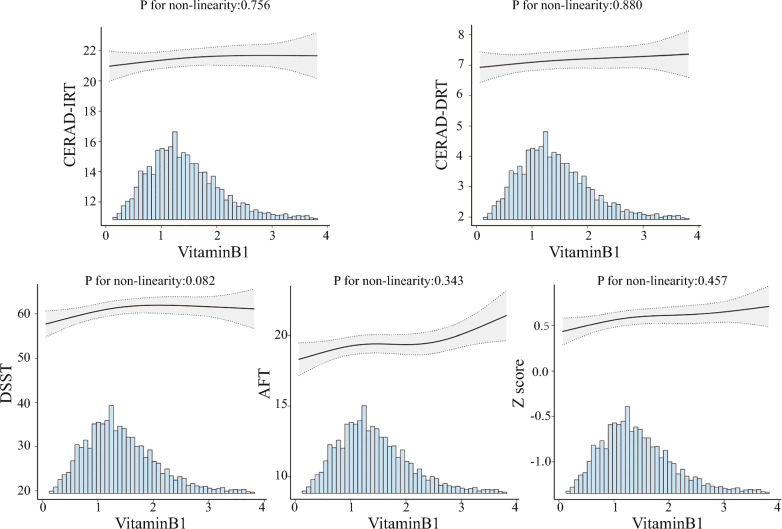
Association between dietary vitamin B1 intake and cognitive performance in DSST, AFT, CERAD-IRT, CERAD-DRT and z score. Solid and dashed lines represent the predicted value and 95% confidence intervals. Adjusted for age, gender, race/ethnicity, poverty income ratio, marital status, education level, body mass index, smoking status, alcohol status, hypertension, diabetes mellitus, cardiovascular disease history and stroke. Only 99% of the data is shown

After fully adjusting, vitamin B1 and cognitive function scores showed a dose–response relationship. In restricted cubic spline models, vitamin B1 was positively associated with each of the cognitive scores in a linear manner (Fig. 2).

### Subgroup analysis

**Fig. 3:**
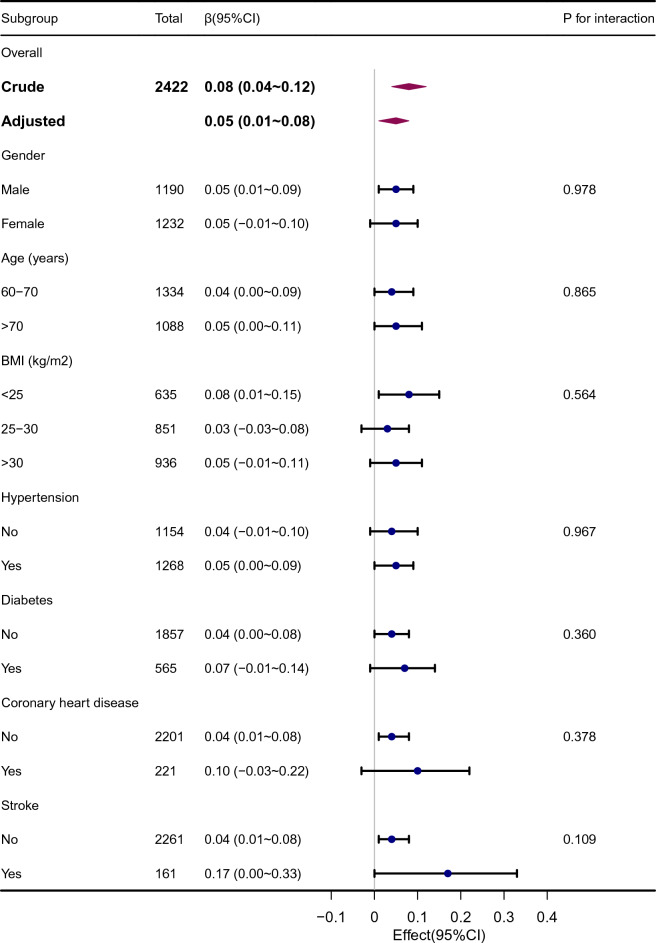
Stratified analyses of the association between cognitive performance and dietary vitamin B1 intake according to baseline characteristics in z score test. The P value for interaction represents the likelihood of interaction between the variable and vitamin B1. *CI* confidence interval

In addition, the study examined possible moderators of the association between cognitive performance and dietary vitamin B1 intake of cognitive scores, including gender (male vs. female), age (60–70 vs. > 80), BMI (< 25 vs. 25–30 vs. > 30 kg/m^2^), hypertension (yes vs. no), diabetes mellitus (yes vs. no), cardiovascular disease history (yes vs. no) and stroke (yes vs. no). The results of the subgroup analysis for the association between dietary vitamin B1 intake and cognitive function are shown in Additional file 2: Table S2. High intake of vitamin B1 was associated with higher global z score, if the participants were without diabetes (β = 0.09, 95% CI 0.01 ~ 0.17; p for interaction = 0.014), and coronary heart disease (β = 0.09, 95% CI 0.02 ~ 0.17; p for interaction = 0.007). Compared with Q1, participants in Q4 groups were found to have a higher globe z score (β = 0.08, 95%CI −0.02 ~ 0.18; p for trend = 0.045) and DSST score (β = 2.4, 95% CI 0.42 ~ 4.38; p for trend = 0.011) in the subgroup of hypertension. No significant interactions were found between dietary vitamin B1 intake and participant characteristics or other disease states (Fig. 3).

## Discussion

This study examined data obtained from a cross-sectional study conducted from 2011 to 2014, specifically targeting the older adult population. Vitamin B1 intake was associated with better cognitive scores in this study using multivariate-adjusted linear regression analysis, which remained significant after adjusting for all potential confounders. Moreover, we observed a dose-dependent association between dietary vitamin B1 consumption and low cognitive performance in US adults (global z score, P for nonlinearity = 0.457).

Several studies have also investigated the relationship between B vitamins and cognition [25, 27, 28]. A meta-analysis has indicated that B vitamin supplementation was associated with decelerating cognitive decline, particularly in populations who have received early and long-term intervention. Additionally, increased dietary folate intake has been associated with a reduced risk of dementia in people without cognitive impairment [9]. In a 4-year follow-up study, vitamin B6 was found to be an important protective factor in maintaining cognitive function in old age, especially in folate and vitamin B12 deficient populations [29]. In a cross-sectional study of 206 Alzheimer's patients, thiamine or its analogs supplementation was found to improve cognitive function [30]. A Chinese study also showed that higher dietary intakes of riboflavin and folate in midlife were associated with a reduced risk of cognitive impairment in old age [31].

Vitamin B1 is an essential nutrient required for optimal cellular functioning. It also was a coenzyme that is essential for the efficient metabolism of carbohydrates, proteins, and fats. Additionally, vitamin B1 plays a crucial part in various brain metabolic processes. By playing a role in oxidation and glucose metabolism, it has also been associated with neurodegenerative diseases [32]. However, the utilization of vitamin B1 decreased in the elderly [12]. Deficiencies of vitamin B1 can result in heart failure and serious neurologic disorders such as paralysis, ataxia, confusion, and delirium [13, 33, 34]. According to Pourhassan et al., the mean level of thiamine in the blood of patients with delirium was significantly lower than that of controls (p = 0.002) [35]. The research conducted by La Rue et al. also revealed a positive association between the consumption of thiamine in one's diet and abstract reasoning abilities [32]. Our study similarly found the protective effect of vitamin B1 on cognitive function. As dietary vitamin B1 intake increased, cognitive scores (including DSST, AFT, and Z score) increased accordingly (p < 0.001).

Although the mechanism of association between vitamin B1 intake and cognitive decline is unclear, we believe our findings are biologically plausible. Previous studies have demonstrated that thiamine deficiency elicits anorexia through the inhibition of the hypothalamic adenosine monophosphate-activated protein kinase signal pathway and disruption of neuroendocrine feedback control over food intake and energy metabolism, which may be related to malnutrition and insufficient thiamine intake in AD patients [36, 37]. Ramamoorthy et al. pointed out the role of neuroinflammation in the dysfunction of thiamin transporters (mediated via transcriptional mechanisms) and suggested optimizing thiamin levels in the brain of AD patients [37]. Vitamin B1 deficiency leads to reduced activity of acetylcholine synthase choline acetyltransferase, and neurogenesis, inducing excessive glutamate release and selective death of the subthalamic midline nucleus, which is involved in brain inflammation and oxidative stress [14].

In this study, we used a multidimensional, comprehensive approach to assess cognitive function, including the CERAD-DRT, CERAD-IRT, DSST, AFT, and z score [38]. Furthermore, confounding variables were adjusted by previous studies and conducting thorough analyses. Additionally, a dose–response analysis was conducted to investigate the potential relationship between vitamin B1 intake and cognitive function. In spite of that, there were several limitations in our study. Firstly, due to the cross-sectional nature of this study, we could not determine the temporal association between vitamin B1 and cognitive function. Additional randomized controlled studies are needed to confirm the findings. Secondly, despite comprehensive adjustments for potential covariates, the presence of reverse causality and residual confounding cannot be entirely ruled out. Certain covariates may possess new indicators that were not considered in this study. Thirdly, as the dietary data for vitamin B1 were collected from self-reported 24-h diet reviews, there might be some measurement and recall errors. Finally, additional research is required to determine whether the current findings can be extrapolated to other populations based on this study of U.S. adults.

## Conclusions

In conclusion, among a representative national sample of US adults, vitamin B1 intake was associated with cognitive performance. Our findings may provide support for further largescale prospective studies to elucidate the exact causality of this relationship.

### Supplementary Information

**Additional file 1: Table S1.** Association of covariates and cognitive function.

**Additional file 2: Table S2.** Association between Dietary Vitamin B1 and Cognitive Function among Different Subgroups.

## Data Availability

Publicly available datasets are available online for this study. The repository/repositories name and accession numbers are available online at http://www.cdc.gov/ nchs/nhanes.htm (accessed on 1 March 2022). Dietary data can be obtained from 2011–2012 Dietary Data—Continuous NHANES (cdc.gov) and 2013–2014 Dietary Data—Continuous NHANES (cdc.gov).

## Abbreviations

NHANES: National Health and Nutrition Examination Survey
NCHS: National Center for Health Statistics
MEC: Mobile Examination Center
DSST: Digit Symbol Substitution Test
AFT: Animal Fluency Test
CERAD: Consortium to Establish a Registry for Alzheimer's disease
CERAD-IRT: Immediate verbal list learning
CERAD-DRT: Delayed verbal list learning
MCI: Mild cognitive impairment
AD: Alzheimer's disease
PIR: Poverty income ratio
Q: Quartile
SD: Standard deviation
IQR: Median and interquartile range
CI: Confidence intervals
