# Supplementary material for: Association between dietary vitamin B1 intake and cognitive function among older adults: a cross-sectional study
Source: J Transl Med. 2024 Feb 16;22:165. doi: 10.1186/s12967-024-04969-3 (PMC10870482; doi:10.1186/s12967-024-04969-3)
Supplement: Supplementary file 1 — Additional file 1: Table S1. Association of covariates and cognitive function. [file 12967_2024_4969_MOESM1_ESM.docx]

Additional Table 1. Association of covariates and cognitive function.

| Variable | DSTT | | AFT | | CERAD-IRT | | CERAD-DRT | | Z score | |
| --- | --- | --- | --- | --- | --- | --- | --- | --- | --- | --- |
|  | β（95%CI） | p-Value | β（95%CI） | p-Value | β（95%CI） | p-Value | β（95%CI） | p-Value | β（95%CI） | p-Value |
| Age (years) | -0.73  (-0.83, -0.64) | <0.001 | -0.17  (-0.20, -0.14) | <0.001 | -0.18  (-0.2, -0.15) | <0.001 | -0.09  (-0.11, -0.08) | <0.001 | -0.04  (-0.04, -0.03) | <0.001 |
| Gender, n (%) |  |  |  |  |  |  |  |  |  |  |
| Male | 1 (reference) |  | 1 (reference) |  | 1 (reference) |  | 1 (reference) |  | 1 (reference) |  |
| Female | 5.15 (3.81,6.49) | <0.001 | -0.23  (-0.67,0.21) | 0.311 | 1.65 (1.29,2.01) | <0.001 | 0.78 (0.60,0.96) | <0.001 | 0.24  (0.18,0.30) | <0.001 |
| BMI (kg/m2) | 0.00  (-0.11,0.10) | 0.94 | 0.02  (-0,01,0.06) | 0.168 | 0.03 (0.00,0.06) | 0.035 | 0.02 (0.01,0.03) | 0.007 | 0.00  (0.00,0.01) | 0.052 |
| Race/ethnicity, n (%) |  |  |  |  |  |  |  |  |  |  |
| Non-Hispanic white | 1 (reference) |  | 1 (reference) |  | 1 (reference) |  | 1 (reference) |  | 1 (reference) |  |
| Non-Hispanic black | -10.55  (-12.18, -8.92) | <0.001 | -3.04  (-3.57, -2.51) | <0.001 | -0.36  (-0.82,0.09) | 0.119 | -0.33  (-0.56, -0.10) | 0.004 | -0.35  (-0.43, -0.27) | <0.001 |
| Mexican American | -10.4  (-12.82, -7.99) | <0.001 | -1.02  (-1.81, -0.23) | 0.011 | -0.82  (-1.50, -0.14) | 0.017 | -0.29  (-0.63,0.05) | 0.092 | -0.28  (-0.39, -0.16) | <0.001 |
| Others | -6.93  (-8.71, -5.14) | <0.001 | -2.76  (-3.34, -2.17) | <0.001 | -0.89  (-1.39, -0.39) | <0.001 | -0.08  (-0.33,0.17) | 0.54 | -0.28  (-0.37, -0.20) | <0.001 |
| Education level (years), n (%) |  |  |  |  |  |  |  |  |  |  |
| <9 | 1 (reference) |  | 1 (reference) |  | 1 (reference) |  | 1 (reference) |  | 1 (reference) |  |
| 9–12 | 16.31 (14.24,18.37) | <0.001 | 1.63 (0.88,2.37) | <0.001 | 2.78 (2.16,3.40) | <0.001 | 1.11 (0.80,1.43) | <0.001 | 0.59  (0.49,0.69) | <0.001 |
| >12 | 27.65 (25.66,29.64) | <0.001 | 4.54 (3.82,5.26) | <0.001 | 4.26 (3.66,4.87) | <0.001 | 1.80 (1.50,2.10) | <0.001 | 1.04  (0.95,1.14) | <0.001 |
| Marital status, n (%) |  |  |  |  |  |  |  |  |  |  |
| Married or living with a partner | 1 (reference) |  | 1 (reference) |  | 1 (reference) |  | 1 (reference) |  | 1 (reference) |  |
| Living alone | -3.52  (-4.89, -2.51) | <0.001 | -0.85  (-1.29, -0.40) | <0.001 | -0.28  (-0.65,0.09) | 0.134 | -0.17  (-0.35,0.02) | 0.078 | -0.12  (-0.19, -0.06) | <0.001 |
| Family income, n (%) |  |  |  |  |  |  |  |  |  |  |
| Low | 1 (reference) |  | 1 (reference) |  | 1 (reference) |  | 1 (reference) |  | 1 (reference) |  |
| Medium | 7.41 (5.87,8.94) | <0.001 | 1.22 (0.69,1.74) | <0.001 | 1.08 (0.64,1.52) | <0.001 | 0.39 (0.17,0.61) | <0.001 | 0.27  (0.19,0.34) | <0.001 |
| High | 16.7 (15.11,18.3) | <0.001 | 3.27 (2.73,3.82) | <0.001 | 2.13 (1.67,2.59) | <0.001 | 0.87 (0.64,1.10) | <0.001 | 0.61  (0.53,0.68) | <0.001 |
| Smoking status, n (%) |  |  |  |  |  |  |  |  |  |  |
| Never | 1 (reference) |  | 1 (reference) |  | 1 (reference) |  | 1 (reference) |  | 1 (reference) |  |
| Former | -1.39  (-2.84,0.06) | 0.061 | 0.24  (-0.23,0.71) | 0.325 | -0.26  (-0.65,0.13) | 0.197 | -0.13  (-0.32,0.07) | 0.202 | -0.04  (-0.10,0.03) | 0.27 |
| Current | -4.48  (-6.63, -2.33) | <0.001 | -0.41  (-1.11,0.29) | 0.253 | -0.24  (-0.82,0.34) | 0.424 | -0.14  (-0.43,0.15) | 0.335 | -0.11  (-0.21, -0.01) | 0.026 |
| Alcohol status, n (%) |  |  |  |  |  |  |  |  |  |  |
| Never | 1 (reference) |  | 1 (reference) |  | 1 (reference) |  | 1 (reference) |  | 1 (reference) |  |
| Former | -0.58  (-2.7,1.55) | 0.595 | 0.61  (-0.08,1.31) | 0.084 | -0.05  (-0.64,0.54) | 0.866 | -0.09  (-0.38,0.20) | 0.553 | 0.01  (-0.09,0.11) | 0.891 |
| Current | 7.75 (5.81,9.69) | <0.001 | 2.49 (1.85,3.12) | <0.001 | 0.89 (0.36,1.43) | 0.001 | 0.42 (0.15,0.68) | 0.002 | 0.32  (0.23,0.41) | <0.001 |
| Hypertension, n (%) |  |  |  |  |  |  |  |  |  |  |
| No | 1 (reference) |  | 1 (reference) |  | 1 (reference) |  | 1 (reference) |  | 1 (reference) |  |
| Yes | -3.2  (-4.55, -1.85) | <0.001 | -1.37  (-1.81, -0.93) | <0.001 | -0.39  (-0.75, -0.02) | 0.037 | -0.17  (-0.35,0.01) | 0.071 | -0.15  (-0.21, -0.09) | <0.001 |
| Diabetes, n (%) |  |  |  |  |  |  |  |  |  |  |
| No | 1 (reference) |  | 1 (reference) |  | 1 (reference) |  | 1 (reference) |  | 1 (reference) |  |
| Yes | -6.82  (-8.40, -5.24) | <0.001 | -1.27  (-1.79, -0.76) | <0.001 | -0.80  (-1.23, -0.37) | <0.001 | -0.48  (-0.69, -0.26) | <0.001 | -0.25  (-0.33, -0.18) | <0.001 |
| Coronary heart disease, n (%) |  |  |  |  |  |  |  |  |  |  |
| No | 1 (reference) |  | 1 (reference) |  | 1 (reference) |  | 1 (reference) |  | 1 (reference) |  |
| Yes | -4.79  (-7.13, -2.44) | < 0.001 | -0.71  (-1.47,0.06) | 0.07 | -1.04  (-1.68, -0.41) | 0.001 | -0.58  (-0.89, -0.26) | <0.001 | -0.22  (-0.33, -0.11) | <0.001 |
| Stroke, n (%) |  |  |  |  |  |  |  |  |  |  |
| No | 1 (reference) |  | 1 (reference) |  | 1 (reference) |  | 1 (reference) |  | 1 (reference) |  |
| Yes | -9.19  (-11.88, -6.5) | <0.001 | -1.77  (-2.65, -0.89) | <0.001 | -1.78  (-2.51, -1.05) | <0.001 | -0.74  (-1.11,-0.38) | <0.001 | -0.39(-0.52,-0.27) | <0.001 |

Abbreviations: %, weighted proportion; OR, odds ratio; CI, confidence interval; BMI, body mass index; DSST, Digit Symbol substation test AFT, Animal Fluency Test; CERAD, Consortium to Establish a Registry for Alzheimer’s disease; CERAD-IRT, immediate recall in CERAD trial; CERAD-DRT, delayed recall in CERAD trial; Z score is average of standardized scores of DSST, AFT, CERAD-IRT, CERAD-DRT; SD, standard deviation.
