# Supplementary material for: Association between dietary vitamin B1 intake and cognitive function among older adults: a cross-sectional study
Source: J Transl Med. 2024 Feb 16;22:165. doi: 10.1186/s12967-024-04969-3 (PMC10870482; doi:10.1186/s12967-024-04969-3)
Supplement: Supplementary file 2 — Additional file 2: Table S2. Association between Dietary Vitamin B1 and Cognitive Function among Different Subgroups. [file 12967_2024_4969_MOESM2_ESM.docx]

Additional Table 2. Association between Dietary Vitamin B1 and Cognitive Function among Different Subgroups

| Variable | DSST | | AFT | | CERAD-IRT | | CERAD-DRT | | Z score | |
| --- | --- | --- | --- | --- | --- | --- | --- | --- | --- | --- |
|  | β (95% CI) | *p*-Value | β (95% CI) | *p*-Value | β (95% CI) | *p*-Value | β (95% CI) | *p*-Value | β (95% CI) | *p*-Value |
| VB1 quartile (mg/day) | |  |  |  |  |  |  |  |  |  |
| Q1(≤0.97) | 1(reference) |  | 1(reference) |  | 1(reference) |  | 1(reference) |  | 1(reference) |  |
| **Gender** |  | 0.548 |  | 0.260 |  | 0.013 |  | 0.203 |  | 0.028 |
| **Male** |  |  |  |  |  |  |  |  |  |  |
| Q2(0.98-1.33) | 0.72  (-1.41~2.85) | 0.507 | -0.26  (-1.16~0.64) | 0.574 | -0.44  (-1.16~0.29) | 0.238 | -0.25  (-0.62~0.13) | 0.200 | -0.05  (-0.16~0.06) | 0.349 |
| Q3(1.34-1.82) | 1.53  (-0.54~3.59) | 0.147 | 0.39  (-0.49~1.26) | 0.386 | 0.38  (-0.33~1.08) | 0.292 | 0 .00  (-0.36~0.37) | 0.981 | 0.06  (-0.04~0.17) | 0.256 |
| Q4(>1.82) | 1.95  (-0.01~3.90) | 0.051 | 0.60  (-0.23~1.42) | 0.159 | 0.50  (-0.17~1.16) | 0.146 | 0.09  (-0.26~0.43) | 0.630 | 0.09  (-0.01~0.19) | 0.071 |
| p for trend |  | 0.037 |  | 0.054 |  | 0.023 |  | 0.277 |  | 0.011 |
| **Female** |  |  |  |  |  |  |  |  |  |  |
| Q2(0.98-1.33) | 2.72 (0.87~4.56) | 0.004 | 0.59  (-0.10~1.28) | 0.094 | 0.35  (-0.27~0.97) | 0.265 | 0.20  (-0.10~0.51) | 0.192 | 0.11 (0.02~0.20) | 0.021 |
| Q3(1.34-1.82) | 2.53 (0.62~4.44) | 0.009 | 0.52  (-0.20~1.23) | 0.157 | 0.82 (0.18~1.46) | 0.012 | 0.32  (0.00~0.63) | 0.050 | 0.14 (0.05~0.23) | 0.004 |
| Q4(>1.82) | 2.11  (-0.13~4.34) | 0.065 | 0.25  (-0.58~1.09) | 0.551 | -0.30  (-1.05~0.45) | 0.435 | 0.11  (-0.26~0.48) | 0.569 | 0.04  (-0.07~0.15) | 0.503 |
| p for trend |  | 0.028 |  | 0.393 |  | 0.694 |  | 0.243 |  | 0.131 |
| **Age (years)** |  | 0.971 |  | 1.000 |  | 0.658 |  | 0.602 |  | 0.807 |
| **60-70** |  |  |  |  |  |  |  |  |  |  |
| Q2(0.98-1.33) | 1.76  (-0.12~3.64) | 0.067 | 0.27  (-0.49~1.03) | 0.487 | -0.13  (-0.74~0.47) | 0.662 | -0.09  (-0.39~0.22) | 0.580 | 0.02  (-0.07~0.11) | 0.645 |
| Q3(1.34-1.82) | 2.16 (0.22~4.10) | 0.029 | 0.47  (-0.32~1.25) | 0.245 | 0.34  (-0.28~0.96) | 0.284 | 0.14  (-0.18~0.45) | 0.385 | 0.09  (-0.01~0.18) | 0.070 |
| Q4(>1.82) | 2.46 (0.51~4.41) | 0.014 | 0.49  (-0.30~1.28) | 0.220 | 0.19  (-0.43~0.82) | 0.546 | 0.19  (-0.13~0.50) | 0.248 | 0.09 (0.00~0.18) | 0.064 |
| p for trend |  | **0.015** |  | 0.192 |  | 0.301 |  | 0.124 |  | 0.030 |
| **>70** |  |  |  |  |  |  |  |  |  |  |
| Q2(0.98-1.33) | 1.98  (-0.04~4.00) | 0.055 | 0.35  (-0.44~1.14) | 0.381 | 0.33  (-0.40~1.06) | 0.375 | 0.19  (-0.18~0.55) | 0.326 | 0.08  (-0.02~0.19) | 0.127 |
| Q3(1.34-1.82) | 1.83  (-0.15~3.81) | 0.071 | 0.52  (-0.25~1.30) | 0.187 | 0.99 (0.28~1.71) | 0.007 | 0.25  (-0.11~0.62) | 0.171 | 0.13 (0.03~0.24) | 0.014 |
| Q4(>1.82) | 1.36  (-0.76~3.47) | 0.210 | 0.48  (-0.34~1.31) | 0.251 | 0.42  (-0.34~1.19) | 0.278 | 0.12  (-0.27~0.51) | 0.543 | 0.08  (-0.03~0.19) | 0.171 |
| p for trend |  | 0.216 |  | 0.207 |  | 0.089 |  | 0.445 |  | 0.092 |
| **BMI (kg/m2)** |  | 0.676 |  | 0.536 |  | 0.887 |  | 0.538 |  | 0.959 |
| **<25** |  |  |  |  |  |  |  |  |  |  |
| Q2(0.98-1.33) | 2.93 (0.13~5.73) | 0.040 | 0.81  (-0.27~1.90) | 0.142 | 0.26  (-0.71~1.24) | 0.600 | 0.22  (-0.25~0.69) | 0.363 | 0.12  (-0.02~0.26) | 0.099 |
| Q3(1.34-1.82) | 2.75  (-0.12~5.61) | 0.061 | 0.50  (-0.61~1.60) | 0.379 | 0.74  (-0.25~1.74) | 0.145 | 0.37  (-0.11~0.85) | 0.129 | 0.14  (0.00~0.29) | 0.049 |
| Q4(>1.82) | 4.13 (1.29~6.97) | 0.004 | 1.14 (0.04~2.24) | 0.042 | 0.03  (-0.96~1.02) | 0.951 | 0.01  (-0.47~0.48) | 0.974 | 0.12  (-0.03~0.26) | 0.113 |
| p for trend |  | **0.008** |  | 0.079 |  | 0.731 |  | 0.828 |  | 0.108 |
| **25-30** |  |  |  |  |  |  |  |  |  |  |
| Q2(0.98-1.33) | 1.06  (-1.33~3.44) | 0.385 | 0.52  (-0.43~1.48) | 0.283 | -0.31  (-1.11~0.50) | 0.456 | 0.05  (-0.37~0.47) | 0.819 | 0.03  (-0.09~0.15) | 0.65 |
| Q3(1.34-1.82) | 2.13  (-0.23~4.49) | 0.077 | 0.57  (-0.38~1.52) | 0.242 | 0.50  (-0.30~1.30) | 0.223 | 0.12  (-0.30~0.53) | 0.581 | 0.10  (-0.02~0.22) | 0.112 |
| Q4(>1.82) | 1.71  (-0.73~4.15) | 0.170 | 0.43  (-0.56~1.41) | 0.394 | 0.32  (-0.50~1.15) | 0.443 | 0.08  (-0.35~0.51) | 0.710 | 0.07  (-0.05~0.19) | 0.260 |
| p for trend |  | 0.117 |  | 0.415 |  | 0.172 |  | 0.653 |  | 0.157 |
| **>30** |  |  |  |  |  |  |  |  |  |  |
| Q2(0.98-1.33) | 2.28 (0.11~4.45) | 0.040 | -0.21  (-1.09~0.66) | 0.632 | 0.26  (-0.45~0.98) | 0.472 | -0.12  (-0.48~0.24) | 0.519 | 0.03  (-0.08~0.13) | 0.654 |
| Q3(1.34-1.82) | 1.87  (-0.32~4.06) | 0.094 | 0.45  (-0.43~1.33) | 0.320 | 0.77 (0.04~1.49) | 0.038 | 0.14  (-0.23~0.50) | 0.467 | 0.10  (-0.01~0.22) | 0.064 |
| Q4(>1.82) | 1.77  (-0.56~4.11) | 0.137 | 0.15  (-0.79~1.09) | 0.755 | 0.53  (-0.24~1.30) | 0.177 | 0.36  (-0.04~0.75) | 0.075 | 0.10  (-0.02~0.22) | 0.094 |
| p for trend |  | 0.162 |  | 0.444 |  | 0.077 |  | 0.044 |  | 0.041 |
| **Hypertension**  **No** |  | 0.244 |  | 0.469 |  | 0.542 |  | 0.165 |  | 0.117 |
| Q2(0.98-1.33) | 3.18 (1.11~5.25) | 0.003 | 0.77  (-0.07~1.62) | 0.074 | 0.40  (-0.28~1.09) | 0.248 | 0.23  (-0.11~0.58) | 0.188 | 0.13 (0.03~0.23) | 0.014 |
| Q3(1.34-1.82) | 2.64 (0.60~4.68) | 0.011 | 0.77  (-0.06~1.61) | 0.07 | 0.88 (0.21~1.56) | 0.010 | 0.31  (-0.03~0.65) | 0.074 | 0.16 (0.05~0.26) | 0.003 |
| Q4(>1.82) | 2.10  (-0.01~4.21) | 0.052 | 0.71  (-0.16~1.58) | 0.108 | 0.38  (-0.32~1.08) | 0.290 | 0.11  (-0.24~0.47) | 0.529 | 0.10  (-0.01~0.20) | 0.072 |
| p for trend |  | 0.093 |  | 0.129 |  | 0.149 |  | 0.459 |  | 0.063 |
| **Yes** |  |  |  |  |  |  |  |  |  |  |
| Q2(0.98-1.33) | 0.97  (-0.91~2.85) | 0.310 | -0.08  (-0.80~0.64) | 0.832 | -0.24  (-0.89~0.40) | 0.459 | -0.15  (-0.47~0.18) | 0.370 | -0.02  (-0.11~0.08) | 0.70 |
| Q3(1.34-1.82) | 1.84  (-0.08~3.76) | 0.061 | 0.25  (-0.49~0.99) | 0.513 | 0.44  (-0.21~1.10) | 0.185 | 0.06  (-0.27~0.39) | 0.736 | 0.07  (-0.03~0.17) | 0.167 |
| Q4(>1.82) | 2.40 (0.42~4.38) | 0.018 | 0.34  (-0.43~1.10) | 0.386 | 0.15  (-0.53~0.83) | 0.664 | 0.19  (-0.15~0.53) | 0.275 | 0.08  (-0.02~0.18) | 0.121 |
| p for trend |  | **0.011** |  | 0.280 |  | 0.288 |  | 0.17 |  | **0.045** |
| **Diabetes** |  | 0.465 |  | 0.572 |  | 0.725 |  | 0.946 |  | 0.824 |
| **No** |  |  |  |  |  |  |  |  |  |  |
| Q2(0.98-1.33) | 2.49 (0.87~4.10) | 0.003 | 0.50  (-0.14~1.15) | 0.128 | -0.10  (-0.64~0.44) | 0.713 | 0.05  (-0.22~0.33) | 0.697 | 0.06  (-0.02~0.14) | 0.144 |
| Q3(1.34-1.82) | 2.50 (0.88~4.12) | 0.003 | 0.63  (-0.01~1.28) | 0.055 | 0.46  (-0.08~1.00) | 0.094 | 0.22  (-0.05~0.50) | 0.111 | 0.11 (0.03~0.20) | 0.005 |
| Q4(>1.82) | 2.31 (0.65~3.97) | 0.006 | 0.61  (-0.05~1.27) | 0.071 | 0.15  (-0.41~0.70) | 0.602 | 0.19  (-0.09~0.47) | 0.188 | 0.09 (0.01~0.17) | 0.032 |
| p for trend |  | **0.010** |  | 0.067 |  | 0.261 |  | 0.106 |  | **0.014** |
| **Yes** |  |  |  |  |  |  |  |  |  |  |
| Q2(0.98-1.33) | 0.29  (-2.46~3.03) | 0.839 | -0.32  (-1.37~0.74) | 0.558 | 0.42  (-0.54~1.38) | 0.394 | -0.07  (-0.55~0.41) | 0.772 | 0 .00  (-0.14~0.15) | 0.946 |
| Q3(1.34-1.82) | 1.12  (-1.64~3.88) | 0.427 | 0.11  (-0.95~1.17) | 0.842 | 0.97 (0.00~1.94) | 0.050 | 0.07  (-0.41~0.55) | 0.775 | 0.08  (-0.06~0.23) | 0.266 |
| Q4(>1.82) | 2.06  (-0.89~5.01) | 0.172 | 0.36  (-0.78~1.49) | 0.537 | 0.56  (-0.47~1.59) | 0.290 | 0.02  (-0.50~0.53) | 0.948 | 0.08  (-0.08~0.23) | 0.317 |
| p for trend |  | 0.143 |  | 0.435 |  | 0.141 |  | 0.804 |  | 0.193 |
| **Coronary heart disease** |  | 0.952 |  | 0.982 |  | 0.347 |  | 0.886 |  | 0.878 |
| **No** |  |  |  |  |  |  |  |  |  |  |
| Q2(0.98-1.33) | 1.87 (0.41~3.33) | 0.012 | 0.26  (-0.32~0.84) | 0.376 | 0.15  (-0.34~0.65) | 0.537 | 0.03  (-0.22~0.27) | 0.839 | 0.05  (-0.02~0.12) | 0.173 |
| Q3(1.34-1.82) | 2.07 (0.60~3.54) | 0.006 | 0.49  (-0.09~1.07) | 0.100 | 0.62 (0.12~1.11) | 0.014 | 0.16  (-0.09~0.40) | 0.219 | 0.10 (0.03~0.18) | 0.006 |
| Q4(>1.82) | 2.26 (0.74~3.79) | 0.004 | 0.56  (-0.05~1.16) | 0.070 | 0.31  (-0.20~0.83) | 0.229 | 0.15  (-0.11~0.40) | 0.257 | 0.09 (0.02~0.17) | 0.018 |
| p for trend |  | **0.004** |  | 0.050 |  | 0.080 |  | 0.161 |  | **0.007** |
| **Yes** |  |  |  |  |  |  |  |  |  |  |
| Q2(0.98-1.33) | 1.55  (-2.95~6.06) | 0.499 | 0.44  (-1.4~2.28) | 0.639 | -1.07  (-2.66~0.52) | 0.190 | 0.00  (-0.90~0.89) | 0.992 | -0.02  (-0.27~0.24) | 0.902 |
| Q3(1.34-1.82) | 2.17  (-2.36~6.70) | 0.349 | 0.40  (-1.45~2.25) | 0.670 | 0.50  (-1.11~2.10) | 0.544 | 0.60  (-0.3~1.51) | 0.191 | 0.14  (-0.11~0.40) | 0.270 |
| Q4(>1.82) | 1.64  (-2.86~6.15) | 0.475 | 0.15  (-1.69~1.99) | 0.876 | -0.15  (-1.75~1.44) | 0.851 | 0.43  (-0.47~1.32) | 0.354 | 0.07  (-0.18~0.32) | 0.593 |
| p for trend |  | 0.473 |  | 0.915 |  | 0.708 |  | 0.217 |  | 0.406 |
| **Stroke** |  | 0.060 |  | 0.070 |  | 0.725 |  | 0.327 |  | 0.116 |
| **No** |  |  |  |  |  |  |  |  |  |  |
| Q2(0.98-1.33) | 1.55 (0.12~2.98) | 0.034 | 0.10  (-0.47~0.68) | 0.216 | 0.00  (-0.48~0.49) | 0.985 | -0.03  (-0.28~0.21) | 0.782 | 0.02  (-0.05~0.10) | 0.515 |
| Q3(1.34-1.82) | 1.97 (0.54~3.40) | 0.007 | 0.36  (-0.21~0.94) | 0.224 | 0.65 (0.16~1.13) | 0.009 | 0.17  (-0.08~0.41) | 0.184 | 0.10 (0.03~0.17) | 0.007 |
| Q4(>1.82) | 1.79 (0.31~3.28) | 0.018 | 0.37  (-0.23~0.96) | 0.152 | 0.31  (-0.19~0.82) | 0.218 | 0.12  (-0.14~0.37) | 0.361 | 0.07 (0.00~0.15) | 0.054 |
| p for trend |  | **0.014** |  | 0.725 |  | 0.045 |  | 0.170 |  | 0.013 |
| **Yes** |  |  |  |  |  |  |  |  |  |  |
| Q2(0.98-1.33) | 7.72 (1.78~13.66) | 0.012 | 3.69 (1.68~5.7) | <0.001 | 0.78  (-1.27~2.84) | 0.458 | 0.82  (-0.12~1.76) | 0.088 | 0.41 (0.11~0.72) | 0.008 |
| Q3(1.34-1.82) | 5.13  (-1.34~11.60) | 0.122 | 2.47 (0.28~4.65) | 0.029 | 0.49  (-1.75~2.73) | 0.67 | 0.38  (-0.65~1.40) | 0.469 | 0.26  (-0.07~0.58) | 0.131 |
| Q4(>1.82) | 9.09 (2.62~15.56) | 0.007 | 3.10 (0.91~5.29) | 0.006 | 0.07  (-2.17~2.31) | 0.952 | 0.88  (-0.14~1.91) | 0.094 | 0.37 (0.04~0.70) | 0.028 |
| p for trend |  | **0.016** |  | **0.017** |  | 0.973 |  | 0.162 |  | 0.056 |

Abbreviations: CI, confidence interval; VB1, vitamin B1; Q1-Q4: Quartile according to vitamin B1 intake; DSST, Digit Symbol substation test AFT, Animal Fluency Test; CERAD, Consortium to Establish a Registry for Alzheimer’s disease; CERAD-IRT, immediate recall in CERAD trial; CERAD-DRT, delayed recall in CERAD trial; Z score is average of the standardized scores of DSST, AFT, CERAD-IRT, CERAD-DRT;
